# Mental health leadership and patient access to care: a public–private initiative in South Africa

**DOI:** 10.1186/s13033-017-0160-4

**Published:** 2017-09-06

**Authors:** Christopher Paul Szabo, Jennifer Fine, Pat Mayers, Shan Naidoo, Tuviah Zabow, Mental Health Leadership Working Group, Jannes Bornman, Francois Petersen, Wendy Vogel, Zaida Damons, Charl Prinsloo, Emily Smith, Elmarie de Lange, Rene Nassen, Anbrenthia Moos, Bernard Janse van Rensburg, Craig Bracken, Salome Mashile, Florence Makobonyane, Zamo Mbele, Jacqui Naude

**Affiliations:** 1 Department of Psychiatry, Faculty of Health Sciences, University of the Witwatersrand, Johannesburg, South Africa; 2 Medical Intelligence and Patient Perspective, Sanofi Global R&D, Paris, France; 3 Division of Nursing and Midwifery, Faculty of Health Sciences, University of Cape Town, Cape Town, South Africa; 4 School of Public Health, Faculty of Health Sciences, University of the Witwatersrand, Johannesburg, South Africa; 5 Department of Psychiatry and Mental Health, University of Cape Town, Cape Town, South Africa

**Keywords:** Mental health, Leadership, South Africa

## Abstract

**Background:**

Mental health leadership is a critical component of patient access to care. More specifically, the ability of mental health professionals to articulate the needs of patients, formulate strategies and engage meaningfully at the appropriate level in pursuit of resources. This is not a skill set routinely taught to mental health professionals.

**Methods:**

A public–private mental health leadership initiative, emanating from a patient access to care programme, was developed with the aim of building leadership capacity within the South African public mental health sector. The express aim was to equip health care professionals with the requisite skills to more effectively advocate for their patients. The initiative involved participants from various sites within South Africa. Inclusion was based on the proposal of an ongoing “project”, i.e. a clinician-initiated service development with a multidisciplinary focus. The projects were varied in nature but all involved identification of and a plan for addressing an aspect of the participants’ daily professional work which negatively impacted on patient care due to unmet needs. Six such projects were included and involved 15 participants, comprising personnel from psychiatry, psychology, occupational therapy and nursing. Each project group was formally mentored as part of the initiative, with mentors being senior professionals with expertise in psychiatry, public health and nursing. The programme design thus provided a unique practical dimension in which skills and learnings were applied to the projects with numerous and diverse outcomes.

**Results:**

Benefits were noted by participants but extended beyond the individuals to the health institutions in which they worked and the patients that they served. Participants acquired both the skills and the confidence which enabled them to sustain the changes that they themselves had initiated in their institutions. The initiative gave impetus to the inclusion of public mental health as part of the curriculum for specialist training.

**Conclusions:**

Despite the significant adverse social and economic costs of mental illness, psychiatric and related services receive a low level of priority within the health care system. Ensuring that mental health receives the recognition and the resources it deserves requires that mental health care professionals become effective advocates through mental health leadership.

## Background

The stigmatization and prejudice experienced by people living with psychiatric disorders remains one of the most pressing challenges in the care of the mentally ill worldwide. Despite the growing awareness of the importance of mental health as a public health and development issue in low and middle income countries, people with psychosocial disabilities continue to be marginalized-socially, occupationally and as beneficiaries of government attention and development aid. Cognizant of the aforementioned issues, the 2014 Declaration on Mental Health in Africa [1] specifically stated that “*There is an urgent need for political vision, commitment, and leadership at the highest level to encourage national dialogue on mental health.*” Such a pronouncement resonates with the notion of developing leadership capacity at a mental health professional level with the intention of promoting enhanced patient care through effective advocacy. In South Africa neuropsychiatric disorders are ranked third in the national burden of disease [2]. It is estimated that one in six South Africans are suffering from a common mental disorder [3] yet mental health is given little attention as a national health priority [4]. The South African National Department of Health (NDoH) itself has acknowledged that “*for the majority of people with mental health problems there is just no care*” [5]. Similar treatment gaps have been noted in many other countries both within and beyond Africa due to low prioritization, poor funding and inadequate human resources for mental health globally [6]. Mental illness contributes to high levels of absenteeism, poor work quality and impaired productivity. However, mental health care continues to be underfunded and under supported. The NDoH currently spends only 4% of its budget (about ZAR 9.3-billion, equivalent to approximately USD 660 million) on mental illnesses. A ministerial level Government official has described mental health as the “*Cinderella of the health system*” [7]. In the Foreword to South Africa’s National Mental Health Policy Framework and Strategic Plan 2013–2020 it is emphasized that good mental health will contribute substantially to economic and social development [8].

Mental health care professionals (MHCPs) are uniquely placed to advocate for their patients. Engaging with hospital administrators, government departments and funders has become an integral part of patient care. Yet in spite of this expectation, MHCPs are ill-prepared to take on an advocacy role. The focus of conventional medical training is generally on individual patient and family interactions. There is little formal training directed at leadership and managerial skills to promote service development, policy planning or building partnerships for action.

Recognizing the potential for mental health care professionals to provide leadership in transforming the health care environment and ensuring better access to care for their patients, the Department of Psychiatry at the University of the Witwatersrand (Johannesburg, South Africa) embarked on an innovative mental health leadership training program conducted in the public mental health sector in collaboration with Sanofi South Africa [9]. A similar leadership and advocacy program has been undertaken in West Africa, i.e. the “Mental Health Leadership and Advocacy Program (mhLAP)” [6]. Although the ultimate goals for the two programs were similar, developing and empowering relevant role players, there were significant differences in approach. Whilst the mhLAP aimed to effect change by promoting the development of advocacy efforts, in the current program an advocacy agenda was embedded in a practical real life project which was pursued within a structured, facilitated process.

Stigma in mental health traditionally focuses on that experienced by patients given the putative impact of stigma on access to care. The focus of the current programme did not have a patient/community orientated approach but rather one of engaging and empowering mental health professionals in the field. The role of stigma in influencing resource allocation would appear to require a broader role for psychiatrists within the context of patient advocacy. Public attitudes towards resource allocation have demonstrated a greater willingness to endorse cuts for spending on psychiatric rather than medical conditions [10]. In addition, mental health care professionals themselves are disempowered by fellow colleagues from outside of psychiatry through denigration [11]. Whilst a recent review of public attitudes appears to show more favourable attitudes towards both psychiatry and psychology [12] disempowerment from within the system needs to be addressed if mental health is to receive the necessary attention and commitment of resources. The specific intention of the current program’s approach was to enhance health care professional leadership skills and enable them to more effectively address the need for mental health to be accorded appropriate priority [13].

## Methods

This is a descriptive report of a process rather than a study per se. The programme did, however generate both quantitative and qualitative data at various longitudinal points which are reported. These relate to evaluation of content and subjective self-assessment.

### Programme

The programme comprised a total of five workshops during 2013 (April, October), 2014 (May, October) and 2015 (March). The first workshop within the program commenced with a formal didactic component in which guest lecturers from academic centres, the National Department of Health, and a patient advocacy group covered an array of topics such as public health, government responsibility and accountability, resource allocation, mental health law and human rights, ethical responsibilities, patient advocacy, professionalism, and leadership and management. Other didactic and motivational components that were introduced at the subsequent workshops included a guest lecture from an experienced clinician who had successfully initiated a project leading to improved clinical services, a facilitated session on a structured approach to problem solving, as well as a qualitative session that focused on self- reflection.

### Participant selection

Entrance into the program was open to all MHCPs in the public sector. An invitation to submit content for consideration was sent to the eight academic Departments of Psychiatry in South Africa. Participants were required to submit a project proposal identifying an unmet need in patient care. A preliminary plan for tackling this was a prerequisite. Hence at the outset it was made clear that participation in the program would require an existing project that would be the focus of ongoing effort. A second requirement was that application to participate had to be made by a team rather than an individual, and the team had to be representative of different areas of mental health expertise.

### Participants

Selected participants included 15 mental health care professionals drawn from six public health care facilities nationally. The participants represented a range of disciplines, i.e. psychiatry, psychiatric nursing, occupational therapy and psychology.

### Projects

Each of the six project groups was assigned to one of three mentors, senior health care professionals with vast experience in the fields of psychiatry, public health and nursing. These mentors provided support, advice and encouragement on a regular and continuous basis. Aside from individual participant/mentor meetings all mentors and participants attended a 6-monthly workshop over a period of 2 years to report on project progress, share successes and tackle challenges. This project design enabled participants to exercise and test their learning in pursuit of a practical objective related to an aspect of their daily work.

The content of the projects was wide ranging covering a number of mental health issues, in both rural and urban settings in South Africa, and in a range of facilities from community clinics to tertiary academic centres.

There was no specific expectation or externally-imposed criteria for success. Each group defined its own aims and objectives and established its own timelines. Where difficulties and obstacles arose these were taken to the group for discussion.

## Results

### Group projects

The inclusion process whereby participants defined their own challenges resulted in a wide range of project content. The titles and objective for each project were as follows:

- Sustainable care pathways in rural mental health-making best use of limited resources [Western Cape] (14). *The project aimed to recruit general doctors to serve in a dedicated mental health clinic in the community setting.*
- Developing an assertive community treatment (PACT) program in the West coast and Winelands region [Western Cape] (15). *The project aimed to reduce admissions.*
- Development of care pathways for some of the common child and adolescent psychiatric disorders and improve flow through the psychiatric services [Western Cape] (16). *The project aimed to improve efficiency.*
- Establishing a multidisciplinary HIV-clinic for adolescents [Western Cape] (17). *The project involved the conceptualization and design of a paediatric HIV mental health screening instrument.*
- Assertive community treatment (ACT) outpatient project [Gauteng] (18). *The project aimed to develop a multidisciplinary group program for the management of patients recovering from their first psychiatric episode.*
- Improving access, referral and service delivery of mental health and psychiatric care in the Johannesburg Metro District [Gauteng] (19). *The project aimed to improve collaboration between a range of sites/services and thus efficiency with improved patient care.*

Detailed accounts from each project leader [14–19] and reflective pieces from each mentor on the process have been previously published [20–22]. What follows is a *selection* of some of the achievements reported by participants during the workshops in relation to *certain* of the projects:

1. *Sustainable Care Pathways in rural mental health*-*making best use of limited resources* [14]. After a false start trying to recruit general doctors to serve in a dedicated mental health clinic in the community setting, this group recognized that, in the absence of additional resources from the Department of Health (Western Cape), they would have to form collaborative partnerships within the local community. They would also need to rely on volunteer community care workers and train them in mental health. One direct outcome measure was the inpatient unit’s re-admission rate (the percentage of the total monthly admissions who are re-admitted within 3 months of being discharged). The group managed to show a significant and sustained improvement, achieving the target set by the Provincial (Western Cape) Department of Health of a re-admission rate of <15%. It must be pointed out that many of the plans were initially unrealistic and overambitious. Even when objectives were not achieved the workshop provided a valuable forum to analyze the reasons for the failure and to initiate new directions.
2. *Develop care pathways for some of the common child and adolescent psychiatric disorders and improve flow through the psychiatric services* [16]. By developing care pathways and ensuring a smooth flow through the service, this project group managed to reduce the waiting list from 6 to 9 months at the start of the training to 4–6 weeks by January 2015. This achievement did not come easily. It required a full review and gap analysis of provincial child and adolescent psychiatric services. This was followed by the design and adoption of referral pathways by participating institutions. Work is ongoing towards the establishment of a single comprehensive database with accurate data collection. Ongoing discussion with stakeholders was essential to the success of this project.
3. *Establishing a multidisciplinary HIV*-*clinic for adolescents* [17]. The most striking achievement of this project group was the conceptualization and design of a paediatric HIV mental health screening instrument. This consists of a cognitive screening tool and user guide. Plans are underway for validation of this tool and its incorporation into the HIV directorate assessment packs in paediatric HIV clinics.
  This project also succeeded in improving resources and strengthening networks. Buy-in was obtained for the establishment of a dedicated paediatric neuropsychiatry clinic at a district hospital, and securing a commitment of staffing of two additional medical officers. The clinic will receive referrals for a wide range of neuropsychiatric problems, including paediatric HIV, and will be supported by a strong partnership with its associated tertiary hospital Child and Adolescent Mental Health outreach initiative.
4. *Assertive community treatment (ACT) outpatient project* [18]. The aim of this project was to develop a multidisciplinary group program for the management of patients recovering from their first psychiatric episode. These patients are at high risk of relapse and re-admission, a phenomenon known as “the revolving door syndrome”. Of the 35 patients who had participated in the ACT groups, 24 completed the course, 5 were well enough to proceed to vocational rehabilitation, and 7 were able to find employment. When reviewing successes at the end of the program the participants noted that over the course of the 2 years they had expanded their staff complement from 2 to 4 clinicians, secured 2 venues and established partnerships with other health facilities, NGOs and the community. They had also established a network of partners.

### Participant ratings and self-evaluation of experience

Intermittent longitudinal evaluation of the programme content as well as participant self evaluation of growth and development over the 2-year duration of the programme generated both qualitative and quantitative data as follows:

### Quantitative

#### Rating of lectures

Following the first workshop participants (n = 15) were requested to rate both individual lectures as well as their overall experience. Most were rated “excellent” with “Leadership” being rated so by 71% of participants and “Government” by 85% (Table 1). This appears to reflect the appreciation, and need, for content on leadership with the opportunity to engage with a senior Government official in terms of what Government ‘hears’ when we ‘speak’. A critical lesson was that clinicians are generally ill equipped to engage meaningfully and that to be heard requires a skill set that is not part of routine training.

**Table 1:** Participant evaluation

Topic	Below average	Average (%)	Good (%)	Excellent (%)
Law			46	54
Ethics			57	43
Patient advocacy			43	57
Leadership			29	71
Human rights			54	46
Government			15	85
Overall usefulness/value		7	29	64

### Qualitative

#### Participant comments

Participants (n = 15) were given an opportunity to reflect on their experience of the program following the *first workshop* in an open ended way and during the *third workshop* during a facilitated reflective session. Based on the comments, three broad domains emerged, i.e. *empowerment* following the first workshop and *personal leadership* and *strategic ability* following the subsequent second and third workshops.

*Empowerment*

Selected comments, congruent with the structured feedback, provide more of a sense of their individual experiences. The overriding sentiment was one of empowerment and optimism through information that addressed issues of relevance.

- *We are definitely more informed as clinicians and managers to know how to pitch recommendations and what factors to focus on when planning, implementing and monitoring projects and programmes.*
- *An excellent initial workshop that left me inspired and well informed about the challenges and opportunities in improving mental health services.*
- *Worth attending and very informative. Look forward to the next workshop.*
- *All presentations were excellent and informative as well as very relevant to areas of service delivery.*
- *We discovered how we need to approach some of the service challenges facing us daily, and how we can learn from others in our endeavors.*
- *This was an excellent opportunity to stimulate thought and conversation.*
- *Thank you for the opportunity to attend. It was inspirational.*
- *Thank you for the stimulating and productive workshop, which has a useful balance between practical considerations and useful information.*

*Personal leadership*

Participants experienced a sense of growth through both exposure to the programme as well their own efforts outside of the workshops. Part of that growth involved greater self-awareness regarding their own attitudes and potential, as well as a better understanding of systems and team work.

- *Attendance of the leadership programme and witnessing the fruition of the project has resulted in huge personal growth in relation to developing leadership skills, project management, and acquiring knowledge in the field of HIV.*
- *It has resulted in huge personal and professional growth. I have first*-*hand experienced the growth of leadership within myself but also the inspiration that leadership can galvanise.*
- *This has been a huge learning curve with regard to navigating different systems and organizational cultures.*
- *Realization of own negative attitudes, lacking in inspiration.*
- *Allow others to take the lead at times.*
- *Appreciate team members’ contribution. Measure the “unseen effort”.*
- *Keeping a positive frame of mind and vision of future is very challenging.*

*Strategy and execution*

Participants had begun to evolve strategically, with a greater sense of refining their aims, networking and thinking beyond their immediate clinical environment. Moreover, they had started to see results.

- *Identify gaps in the system.*
- *Getting to know how “things” work in a macro context.*
- *Development of managerial skills.*
- *How to develop a program, identify positive aspects and adapt according to patient need.*
- *Learning how to network/engage with different role players.*
- *Importance of simplicity.*
- *Value of strong collaboration with multidisciplinary team.*
- *This has been a huge learning curve with regard to navigating different systems and organizational cultures. I have compiled the budget and participated in maintaining financial control during the purchase of the contents of the toolkit.*
- *Despite the challenges we have actually reached completion and stayed ‘on track’. Our time management has been excellent as we set ourselves manageable tasks, with an action list which was updated regularly at planning meetings.*
- *Seeing all the projects grow from uncertainty (“is it possible?”) to well*-*constructed objectives in 2013; and to specific achievable objectives in 2014 with positive outcomes*.

### Impact on public mental health training in South Africa

As the leadership training project gained momentum and with increasing recognition and acknowledgement, public mental health, leadership and management skills were introduced into the formal curriculum of the national specialist psychiatry qualification, i.e. the Fellow of the College of Psychiatrists (FCPsych) which is under the auspices of the Colleges of medicine of South Africa [https://www.cmsa.co.za].

A training symposium in public mental health was subsequently held in October 2015. This national symposium was a direct outcome of the mental health leadership program. Supported by Sanofi, it was hosted by the University of the Witwatersrand, in partnership with the College of Psychiatrists (the national body overseeing specialist training in South Africa) and the South African Society of Psychiatrists (SASOP), attesting to the collaboration of the academic teaching, government, professional associations and industry in pursuing this initiative.

Invited speakers were from the disciplines of Psychiatry, Public Mental Health and Public Health. They represented a number of universities nationally and spoke on a range of curriculum related topics, specifically: *Public Health and Public Mental Health, Epidemiology, Mental Health policy and Service Organization, Poverty and Social Determinants of Mental Health, Advocacy and Rehabilitation, Disability and Incapacity Assessment* and *Leadership*. Invited specialists in training (n = 38) were in their 3rd year of training noting that 4 years is the required minimum period. They were drawn from Departments of Psychiatry from 7 of the 8 medical schools nationally. Attendees (n = 20) rated the lectures between 3.35 to 3.8 out of a maximum of 4 with the overall usefulness of the event rated 3.55 out of a possible 4. A range of specific comments amplified the noted scores.

It is planned to make this a bi-annual educational event with the next public mental health Forum scheduled for September 2017.

## Discussion

This program was designed to equip mental HCPs with the leadership and managerial skills to better enable them to advocate for their patients. It offered a novel, collective and supportive environment outside of the isolation of the workplace in which the execution of concrete plans with defined objectives could be discussed and analysed. Based on individual comments and project outcomes it appears that the programme objectives were achieved. Skills have been acquired, confidence built and leadership roles undertaken.

Participants are now extensively networked both internally and externally. They have established relationships with a number of health professional colleagues in numerous locations. They have learnt to engage more successfully with administrators and decision makers within their institutions and referring health care facilities. As permanent employees in the state health sector, they will continue to utilize these skills in effecting change to the broader benefit of the health services and their patients.

The program has done far more than equipping individuals. The six projects themselves have achieved considerable success. Systems have been improved and additional resources drawn in. Stakeholders have been engaged and partnerships forged. Changes have been embedded and have grown endogenously in each participating health district. The momentum is now independent of the individuals who initiated the changes.

From a financial perspective, the projects have been self- sustaining with no need for the commitment of additional budgetary resources. By targeting MHCPs already inserted into the health services, and tackling participant-identified real-life situations as practical training projects, there have been minimal ongoing costs, and no need for additional staffing support. As a training initiative the concept is transferable. Participants have identified local needs and structural constraints, ensuring that the content is locally relevant.

The program has been impactful and widely acknowledged, both locally and internationally following presentations in various forums, most recently at the World Psychiatric Association International Conference 2016 in Cape Town, South Africa and the 2016 World Association for Social Psychiatry Congress in New Delhi, India. The need for a public mental health agenda within South African psychiatry has repeatedly been raised [23, 24]. The inclusion of public mental health leadership training in the specialist training curriculum of the College of Psychiatrists, a direct outcome of the Mental Health Leadership Training Programme, goes some way to addressing this with a clear intention to broaden the pool of health care professionals who are better equipped to engage in patient advocacy.

As this was an educational and empowerment programme rather than a study, it is difficult to exclude the possibility that at least some of the activities and personal development that we have documented would not have occurred independently, or as a result of some other intervention. We can say with confidence, however, that the programme was a catalyst to the projects themselves, as entry was based on the generation of a project idea. We have to assume that the successful execution thereof was at least in part a consequence of the mentor supervision, and intermittent workshops designed to analyse successes and overcome difficulties. The very enthusiastic ratings and qualitative responses expressed by individual team project members (see “Results” section) indicates that they themselves attributed their enhanced skills and leadership development to the initiative itself. The value that the participants attributed to the programme is reflected in the remarkable achievement of 100% attendance at each of the five 6-monthly workshops extending over a period of 2 years.

To the extent that the selection process—via academic teaching institutions and their satellite services—targeted motivated, committed individuals, the outcomes may not be generalizable to clinicians in the discipline generally. However, it is anticipated that the introduction of public mental health into specialist training will enhance the awareness, capacity and inclination of the broader mental health care community to pursue similar initiatives.

## Conclusion

The aim of the programme was to build leadership capacity amongst MHCPs in a resource limited setting. It was a clinician-driven, patient-focused, mentor-supported training program, driven by a public–private partnership. The process took a number of years from conception and development (2011, 2012) to implementation (2013, 2014), culminating in the integration of public mental health into the specialist training curriculum and a national Public Mental Health Forum in 2015. A further Forum is scheduled for 2017 with the intention of this being a 2 yearly event. The programme has been successful both at the individual and organizational level. Mental health care professionals have been empowered, increased resources have been secured and greater efficiencies introduced into the health system. We now have 15 mental health clinician advocates in multiple locations who are potential drivers of change in the health sector.

The outcomes went well beyond personal development, improved services and better resources, ultimately influencing national public mental health education and training through curriculum development of specialist training. The Mental Health Leadership Programme has been the catalyst for training of emerging psychiatry specialists in public sector advocacy. These graduates will now be better equipped to drive change in the public mental health sector. The net result should, in time, see enhanced services and better resource allocation for the vast numbers of patients with mental illness in South Africa.
